# Impact of Contrast Agent Viscosity and Fallopian Tube Inner Diameter on Tubal Visualization in MR Hysterosalpingography: A Phantom Study

**DOI:** 10.2174/0115734056397661251203065719

**Published:** 2026-02-19

**Authors:** Jin Ding, Lei Shan, Huanzhi Ding, Peng Wu, Yanyu Zhong, Ximing Wang

**Affiliations:** 1 Department of Obstetrics and Gynecology Ultrasound, The First Affiliated Hospital of Soochow University, Suzhou City, China; 2 Department of Radiology, The First Affiliated Hospital of Soochow University, Suzhou City, China; 3 Shanghai Philips Healthcare Company, Shanghai, China; 4 Reproductive Medicine Center, The First Affiliated Hospital of Soochow University, Suzhou City, China

**Keywords:** Magnetic resonance imaging, Hysterosalpingography, Contrast media, Fallopian tubes, Phantom models, Image quality assessment

## Abstract

**Introduction:**

MR-HSG offers radiation-free multiplanar visualization for evaluating fallopian tube patency, but persistent challenges in achieving consistent high-quality visualization, particularly in smaller-diameter segments, limit clinical adoption. Previous studies modifying contrast viscosity achieved only partial improvements. This study aimed to use standardized phantom models simulating the female pelvis to systematically explore factors affecting MR-HSG image quality and provide evidence-based guidance for protocol optimization.

**Method:**

Nine standardized phantoms were constructed using agar gel with embedded tubes of three inner diameters (4mm, 2mm, and 1.4mm) representing ampullary, isthmic, and interstitial segments. Three contrast agents with different viscosities were tested: gadolinium-saline (2.6 mPa·s), gadolinium-iopromide (7.9 mPa·s), and gadolinium-iodixanol (8.7 mPa·s) at 37°C. T1-weighted 3D-mDIXON sequences with keyhole technology were employed on a 1.5T MRI scanner. Signal intensity measurements and qualitative assessment (good/fair/poor) were performed by blinded evaluators.

**Results:**

No significant differences in signal intensity were found between contrast agents of different viscosities (P>0.05). However, tube diameter significantly affected imaging quality (*P*<0.001). The 4 mm tubes showed the highest SI (5554.49±1042) with 100% good imaging, the 2 mm tubes showed intermediate SI (733.65±78.76) with 100% fair imaging, and the 1.4 mm tubes showed the lowest SI (444.55±34.70) with 100% poor imaging across all contrast agents.

**Discussion:**

Fallopian tube inner diameter is the primary determinant of MR-HSG imaging quality, while contrast agent viscosity (within 2.6-8.7 mPa·s range) shows no significant effect under controlled conditions. This study provides foundational data for understanding physical factors affecting MR-HSG quality and suggests that anatomical factors may be more critical than contrast properties for clinical protocol optimization, potentially reducing procedure costs while maintaining diagnostic quality.

**Conclusion:**

Optimizing spatial resolution to minimize partial volume effects may be more beneficial than modifying contrast agent properties for improving visualization of narrow fallopian tube segments. Clinical validation studies are warranted to confirm these findings in the complex *in vivo* environment.

## 1 INTRODUCTION

Infertility, defined by the World Health Organization as the inability to achieve a clinical pregnancy after 12 months of regular, unprotected sexual intercourse, affects 8-12% of couples of reproductive age globally [1, 2]. Among the various causes of female infertility, fallopian tube pathology accounts for 30-40% of cases, making accurate assessment of tubal patency crucial for diagnosis and treatment planning [3].

Current first-line diagnostic modalities for evaluating fallopian tube patency include X-ray hysterosalpingography (HSG) and ultrasound hysterosalpingo-contrast sonography (HyCoSy). However, each has inherent limitations: HSG exposes patients to ionizing radiation and provides limited extraluminal information [4], while HyCoSy's diagnostic accuracy is highly operator-dependent and offers a restricted field of view [5]. These limitations have driven the search for alternative imaging approaches that can provide compreh-ensive, reproducible assessments without radiation exposure.

Magnetic resonance hysterosalpingography (MR-HSG) has emerged as a promising diagnostic tool, offering detailed multiplanar visualization of the entire female reproductive system, including the uterus, fallopian tubes, ovaries, and surrounding pelvic structures [6, 7]. MR-HSG combines the advantages of excellent soft tissue contrast, lack of ionizing radiation, and three-dimensional imaging capabilities. Despite these advantages, clinical adoption has been limited by persistent challenges in achieving consistent, high-quality visualization of the fallopian tubes, particularly the smaller-diameter segments.

Previous studies have reported that approximately 82% of *in vivo* MR-HSG procedures use gadolinium diluted with saline as the contrast agent [8-13]. However, fallopian tube visualization remains suboptimal, particularly in smaller-diameter segments. To address this limitation, researchers have explored modifying contrast agent properties. Unterweger *et al*. [14] and Winter *et al*. [15] increased contrast viscosity by mixing gadolinium with polyvidone, while Duan *et al*. [16] combined gadolinium with iohexol. These modifications improved visualization rates to 85-90%, yet the narrower interstitial and isthmic portions remained inadequately visualized in many cases.

The relationship among contrast agent properties, anatomical factors, and imaging quality in MR-HSG remains incompletely understood. While clinical studies provide valuable real-world data, the complex interplay of physiological variables (peristalsis, absorption, patient motion) makes it difficult to isolate specific factors affecting image quality. Phantom studies offer a controlled environment to systematically evaluate individual variables without confounding physiological factors.

This study aimed to use standardized phantom models to investigate two key variables affecting MR-HSG image quality: (1) contrast agent viscosity and (2) fallopian tube inner diameter. By isolating these factors in a controlled experimental setting, we sought to determine their relative contributions to imaging quality and provide evidence-based guidance for optimizing clinical MR-HSG protocols.

## 2 METHODS

### 2.1 Study Design

This experimental study employed a randomized, controlled design using standardized phantom models. The study protocol was developed according to established phantom imaging research guidelines. Nine identical phantoms were constructed and randomly allocated to three contrast agent groups using computer-generated randomization (random.org). Allocation concealment was maintained until contrast injection. Image acquisition and analysis were performed under blinded conditions, with evaluators unaware of contrast agent assignments. The study design allowed for systematic evaluation of two independent variables: contrast agent viscosity (three levels) and tube diameter (three levels) in a 3×3 factorial design.

### 2.2 Contrast Agent Preparation and Characterization

Three contrast agent mixtures were prepared 30 minutes before imaging to ensure stability: (1) Gd-NS: Gadoterate meglumine (Gd-DOTA, Jiangsu Hengrui Pharmaceuticals Co., Ltd.) diluted 1:80 with 0.9% sodium chloride solution; (2) Gd-I370: Gd-DOTA diluted 1:300 with iopromide injection (370 mgI/100ml, Bayer Vital GmbH); and (3) Gd-I320: Gd-DOTA diluted 1:300 with iodixanol injection (320 mgI/100ml, Yangtze River Pharmaceutical Group Co., Ltd.). Mixing ratios were determined through preliminary optimization experiments, identifying concentrations providing maximum signal intensity at 1.5T while maintaining clinically feasible injection pressures (Fig. **1**). All solutions were prepared using sterile technique in a laminar flow hood.

Dynamic viscosity measurements were performed using a calibrated rotational viscometer (Brookfield DV2T, USA) with cone-plate geometry. Measurements were conducted at shear rates of 100 s^−1^, approximating physiological flow conditions. Each sample underwent triplicate measurements at both room temperature (20°C) and body temperature (37°C). Temperature control was maintained using a circulating water bath with ±0.1°C precision. The measured viscosities at 37°C were as follows: Gd-NS: 2.6±0.1 mPa·s; Gd-I370: 7.9±0.2 mPa·s; Gd-I320: 8.7±0.3 mPa·s, representing a clinically relevant viscosity range (Table **1**).

### 2.3 Phantom Construction

Phantoms were designed to simulate relevant anatomical and physical properties of the female pelvis. Each phantom consisted of (1) tissue-mimicking medium using agar gel (3% w/v) prepared by dissolving 50g experimental-grade agar powder in 1665ml purified water, heated to 95°C with continuous stirring until completely dissolved; and (2) simulated fallopian tubes using medical-grade polyvinyl chloride infusion tubes with inner diameters of 4 mm (representing ampullary segment, actual range 5-6 mm), 2 mm (representing isthmic segment, actual range 2-3 mm), and 1.4 mm (representing interstitial segment, actual range 0.5-1.0 mm). These diameters were selected based on anatomical measurements from cadaveric studies, representing the range encountered clinically [17]. Each tube was bent into a physiological U-shape configuration mimicking fallopian tube tortuosity and fixed in position using a custom acrylic frame before agar solidification. Phantoms were cast in standardized plastic containers (19.5×13.5×10 cm) to ensure consistent geometry (Fig. **2**). Each phantom contained three tubes with different inner diameters arranged in parallel within the agar matrix. Total preparation yielded nine identical phantoms (three diameters × three replicates).

### 2.4 MRI Protocol and Image Acquisition

Imaging was performed on a 1.5T clinical MRI scanner (Ambition, Philips Healthcare, Best, Netherlands) using a 28-channel phased-array body coil. All phantoms were equilibrated to 37°C in a temperature-controlled water bath for 30 minutes before imaging to simulate physiological conditions. The imaging protocol consisted of (1) localizer sequences using three-plane gradient echo for positioning; (2) baseline T1-weighted imaging for background signal assessment; and (3) dynamic contrast-enhanced sequence using 3D-mDIXON with keyhole technology. The dynamic sequence parameters were as follows: coronal orientation, field of view 250×250×250 mm^3^, acquired voxel size 1×1.5×1.5 mm^3^, reconstructed voxel size 0.5×0.5×0.75 mm^3^, TE1/TE2/TR 2.0/4.1/6.2 ms, flip angle 15°, keyhole percentage 35%, temporal resolution 5.2 seconds, and total acquisition time 3 minutes 38 seconds.

Before contrast injection, tubes were flushed with 5ml saline to eliminate air bubbles. Contrast injection began 5 seconds after sequence initiation. A single experienced operator manually injected 10ml of assigned contrast agent at a controlled rate of 0.11±0.01 ml/s using a calibrated syringe pump. Injection pressure was monitored using an inline pressure transducer. Each combination of tube diameter and contrast agent underwent three independent acquisitions.

### 2.5 Image Analysis

#### 2.5.1 Qualitative Assessment

Maximum intensity projection (MIP) reconstructions were generated using Philips IntelliSpace Portal by a radiologic technologist with 12 years of experience. A standardized viewing protocol was established: window width 500, window level 250, slice thickness 10mm with 5mm overlap. Image quality was assessed using a predefined three-tier scoring system: (1) good - homogeneous high signal throughout entire tube length, sharp margins, no signal voids; (2) fair - inhomogeneous signal with areas of reduced intensity, partially blurred margins, <25% signal voids; and (3) poor - predominantly low signal, indistinct margins, >25% signal voids. Two board-certified radiologists with 10 and 11 years of experience independently scored all images in randomized order. Readers were blinded to contrast agent type and provided with standardized reference images for each quality tier. Inter-rater reliability was assessed using weighted kappa statistics (κ=0.82, indicating excellent agreement). Discrepancies were resolved through consensus review.

#### 2.5.2 Quantitative Assessment

Signal intensity measurements were performed by a radiologist with 11 years of experience using standardized regions of interest (ROIs). Circular ROIs (5mm diameter) were placed at three anatomically relevant locations: (1) proximal segment (5mm from tube entrance); (2) middle segment (midpoint of tube); and (3) distal segment (5mm from tube exit). Each measurement was repeated three times with ROI repositioning. The background signal was measured in adjacent agar. Signal-to-noise ratio (SNR) was calculated as follows: SNR = SItube/Sdbackground, while contrast-to-noise ratio (CNR) was calculated as follows: CNR = (SItube - SIagar)/SDbackground.

### 2.6 Statistical Analysis

Statistical analyses were performed using SAS 9.4 (SAS Institute, Cary, NC). Data distribution was assessed using Shapiro-Wilk tests and Q-Q plots. For normally distributed data with equal variances (Levene's test), one-way ANOVA was used for group comparisons, followed by Student-Newman-Keuls post-hoc testing. For non-normally distributed data, Kruskal-Wallis tests were employed with Dunn's post-hoc correction.

Two-way ANOVA was used to evaluate the interaction between contrast viscosity and tube diameter. Effect sizes were calculated using eta-squared (η^2^). Inter-rater reliability was assessed using weighted kappa statistics. All tests were two-sided with significance set at *P*<0.05.

As this was an exploratory phantom study without prior effect size data, formal sample size calculation was not performed. The study was designed to detect large effect sizes (Cohen's d>0.8) with 80% power, requiring n=3 per group. The results are presented as mean±standard deviation unless otherwise specified.

## 3 RESULTS

### 3.1 Contrast Agent Properties and Injection Characteristics

Viscosity measurements demonstrated a 3.3-fold difference between the lowest (Gd-NS: 2.6±0.1 mPa·s) and highest viscosity (Gd-I320: 8.7±0.3 mPa·s) agents at physiological temperature. Temperature significantly affected viscosity, with all agents showing 35-45% higher viscosity at 20°C compared to 37°C temperature (Table **1**).

Injection resistance varied significantly with tube diameter (*P*<0.001). The 4mm tubes required minimal injection pressure (8.2±1.3 kPa), while 2mm tubes required moderate pressure (24.7±3.1 kPa), and 1.4mm tubes required the highest pressure (58.3±5.2 kPa), resulting in partial contrast spillage in 2 of 9 injections. No significant difference in injection pressure was observed between contrast agents within the same tube diameter (*P*=0.43).

### 3.2 Qualitative Imaging Assessment

Dynamic imaging successfully captured contrast progression through all tubes (Fig. **3**). Complete tube opacification occurred at different time points: 4mm tubes at 40±3 seconds, 2mm tubes at 50±5 seconds, and 1.4mm tubes at 60±8 seconds. Image quality assessment revealed consistent patterns across all contrast agents (Fig. **4**): (1) 4mm tubes achieved 100% “good” quality rating (9/9 phantoms); (2) 2mm tubes achieved 100% “fair” quality rating (9/9 phantoms); and (3) 1.4mm tubes achieved 100% “poor” quality rating (9/9 phantoms). No significant differences in qualitative scores were observed between contrast agents for any tube diameter (*P*=0.91). Inter-rater agreement was excellent (κ=0.82, 95% CI: 0.74-0.90).

### 3.3 Quantitative Signal Analysis

Signal intensity measurements revealed significant effects of tube diameter but not contrast viscosity (Tables **2** and **3**). Highly significant differences were observed between tube diameters (F=384.55, *P*<0.001, η^2^=0.89), with 4mm tubes showing SI of 5554.49±1042, 2mm tubes showing 733.65±78.76, and 1.4mm tubes showing 444.55±34.70 (Fig. **5**). Post-hoc analysis confirmed significant differences between all diameter pairs (*P*<0.001 for all comparisons). In contrast, no significant differences were found between contrast agents within any tube diameter: 4mm tubes (*P*=0.96), 2mm tubes (*P*=0.88), and 1.4mm tubes (*P*=0.92). Signal-to-noise ratio (SNR) and contrast-to-noise ratio (CNR) both decreased proportionally with tube diameter, with SNR values of 42.3±8.1, 12.4±2.3, and 7.8±1.2 for 4mm, 2mm, and 1.4mm tubes, respectively (Fig. **6**). Two-way ANOVA revealed no significant interaction between tube diameter and contrast viscosity (F=0.18, *P*=0.95), confirming independent effects.

### 3.4 Regional Signal Variations

Analysis of signal distribution along tube length revealed diameter-dependent patterns. The 4mm tubes showed uniform signal throughout (coefficient of variation <5%), the 2mm tubes demonstrated a mild signal decrease distally (proximal: 756±81, distal: 698±75, *P*=0.03), and the 1.4mm tubes exhibited a significant signal decrease distally (proximal: 478±39, distal: 389±31, *P*<0.001).

## 4 DISCUSSION

This controlled phantom study provided systematic evidence that fallopian tube diameter, rather than contrast agent viscosity, is the primary determinant of imaging quality in MR-HSG. Our findings have important implications for understanding the persistent challenges in clinical MR-HSG and for developing optimization strategies.

The dominant effect of tube diameter on imaging quality can be explained by fundamental MRI physics principles. Signal intensity in MRI is proportional to the number of excited protons within a voxel. With our voxel size of 1×1.5×1.5mm^3^, the 1.4mm diameter tubes experienced significant partial volume effects, where tube contents occupied only ~30% of voxel volume, with the remainder filled by surrounding agar. The signal intensity decreased disproportionately with tube diameter due to partial volume effects, with 4mm tubes showing 12.5-fold higher signal than 1.4mm tubes. The lack of viscosity effect contradicted previous clinical studies [14, 15], but was consistent with MRI physics. In our controlled environment without absorption or peristalsis, viscosity does not affect proton density or relaxation times within the clinical range tested (2.6-8.7 mPa·s). The discrepancy with clinical studies likely reflects the complex *in vivo* environment where higher viscosity may reduce contrast absorption and slow clearance, indirectly improving visualization.

Our findings have suggested several strategies for optimizing clinical MR-HSG. First, sequence optimization for smaller tube segments could involve reducing voxel size below 1mm^3^ to significantly improve visualization, though at the cost of increased scan time and reduced SNR. Second, within the viscosity range tested, contrast choice can be based on safety and cost considerations rather than viscosity alone, as the 3-fold viscosity difference showed no imaging benefit in static conditions. Third, higher injection pressures may be necessary for complete opacification of narrow segments, but must be balanced against patient comfort and risk of tubal spasm. Fourth, expectations should be adjusted based on anatomical segment, with interstitial portions inherently more challenging to visualize.

Our results have been found to align with the anatomical distribution of visualization success in clinical MR-HSG studies, where ampullary segments (5-6mm diameter) are visualized in >95% of cases, while interstitial segments (<1mm) are seen in only 40-60% [10, 11, 16]. However, our finding that viscosity does not affect imaging quality in controlled conditions differs from clinical reports. This discrepancy highlights the importance of distinguishing between direct physical effects (studied here) and indirect physiological effects (absorption, clearance, peristalsis) that occur clinically. The complete visualization achieved even in 1.4mm tubes, albeit with poor quality, suggests that technical factors rather than fundamental physical limitations explain the non-visualization of small segments clinically. Bowel gas artifacts, motion, and tubal spasm likely contribute more to failed visualization than contrast properties alone.

This study's strengths include the controlled experimental design that allowed isolation of specific variables without physiological confounders, standardization through identical phantom construction and temperature control, blinded assessment to reduce observer bias, comprehensive evaluation using both quantitative and qualitative measures, and clinical relevance with tube diameters and viscosities matching clinical scenarios. The reproducibility of our findings across multiple phantoms and contrast agents strengthens the validity of our conclusions.

Several limitations must be acknowledged. Our static model lacked physiological dynamics, including peristalsis, absorption, and mucus interaction, which may affect contrast distribution clinically. The simplified anatomy using straight tubes cannot replicate the complex tortuosity, mucosal folds, and variable luminal diameter of actual fallopian tubes. Though clinically relevant, testing more extreme viscosities might reveal threshold effects. Results at 1.5T may not directly translate to 3T systems, where susceptibility artifacts differ. While adequate for detecting large effects, subtle differences may have been missed due to our sample size. Additionally, as this was an exploratory study without prior effect size data, a formal sample size calculation was not performed.

Future studies should develop dynamic phantoms incorporating pulsatile flow to better simulate physiological conditions. Testing additional contrast agents, including experimental high-relaxivity agents, may identify formulations that could overcome current limitations. An evaluation of advanced sequences, such as compressed sensing and parallel imaging, could improve spatial resolution without prohibitive time penalties [18, 19]. Most importantly, correlating phantom findings with patient outcomes may establish the clinical relevance of our observations. An investigation of temperature effects on contrast distribution may also provide insights, as we observed significant viscosity changes between room and body temperature.

Recent advances in contrast-enhanced MRI have shown promise for improving visualization [20]. Studies on contrast media viscosity effects in renal imaging have demonstrated that viscosity can affect hemodynamics and tissue perfusion [21, 22], suggesting that our phantom findings may not fully capture the complex *in vivo* interactions. However, these studies have also raised safety concerns about high-viscosity agents, particularly regarding contrast-induced nephropathy [21, 22], emphasizing the need for careful benefit-risk assessment when considering viscosity modifications. Despite these technical considerations, MR-HSG combined with comprehensive pelvic MRI has demonstrated clinical value in the diagnostic workup of female infertility [23].

While not directly assessed, our findings have important cost implications. If viscosity does not improve imaging in static conditions, less expensive and lower-viscosity agents may be preferred. However, comprehensive cost-effectiveness analysis must consider patient comfort, procedure time, and diagnostic accuracy in clinical settings. The potential to optimize protocols based on anatomical considerations rather than expensive contrast modifications could significantly reduce procedure costs while maintaining diagnostic quality.

## CONCLUSION

This phantom study has demonstrated fallopian tube inner diameter as the primary determinant of MR-HSG imaging quality, with smaller diameters showing proportionally reduced signal intensity independent of contrast agent viscosity within the tested range (2.6-8.7 mPa·s at 37°C). These findings suggest that optimizing spatial resolution and minimizing partial volume effects through advanced imaging sequences may be more beneficial than modifying contrast agent properties for improving visualization of narrow fallopian tube segments. While phantom studies could not fully replicate the complex *in vivo* environment, our results have provided essential baseline data for understanding the physical factors affecting MR-HSG image quality and can guide future protocol optimization efforts. Clinical validation studies are warranted to confirm these findings and develop evidence-based protocols for MR-HSG in infertility evaluation.

## AUTHORS' CONTRIBUTIONS

The authors confirm their contributions to the paper as follows: W. X.M. and Z.Y.Y.: Conceived the study; D.J., S.L., D. H.Z., and W.P.: Participated in its design, data analysis, and statistical analysis; D.J. and S.L.: Helped in drafting the manuscript. All authors read and approved the final manuscript.

## Figures and Tables

**Fig. (1):**
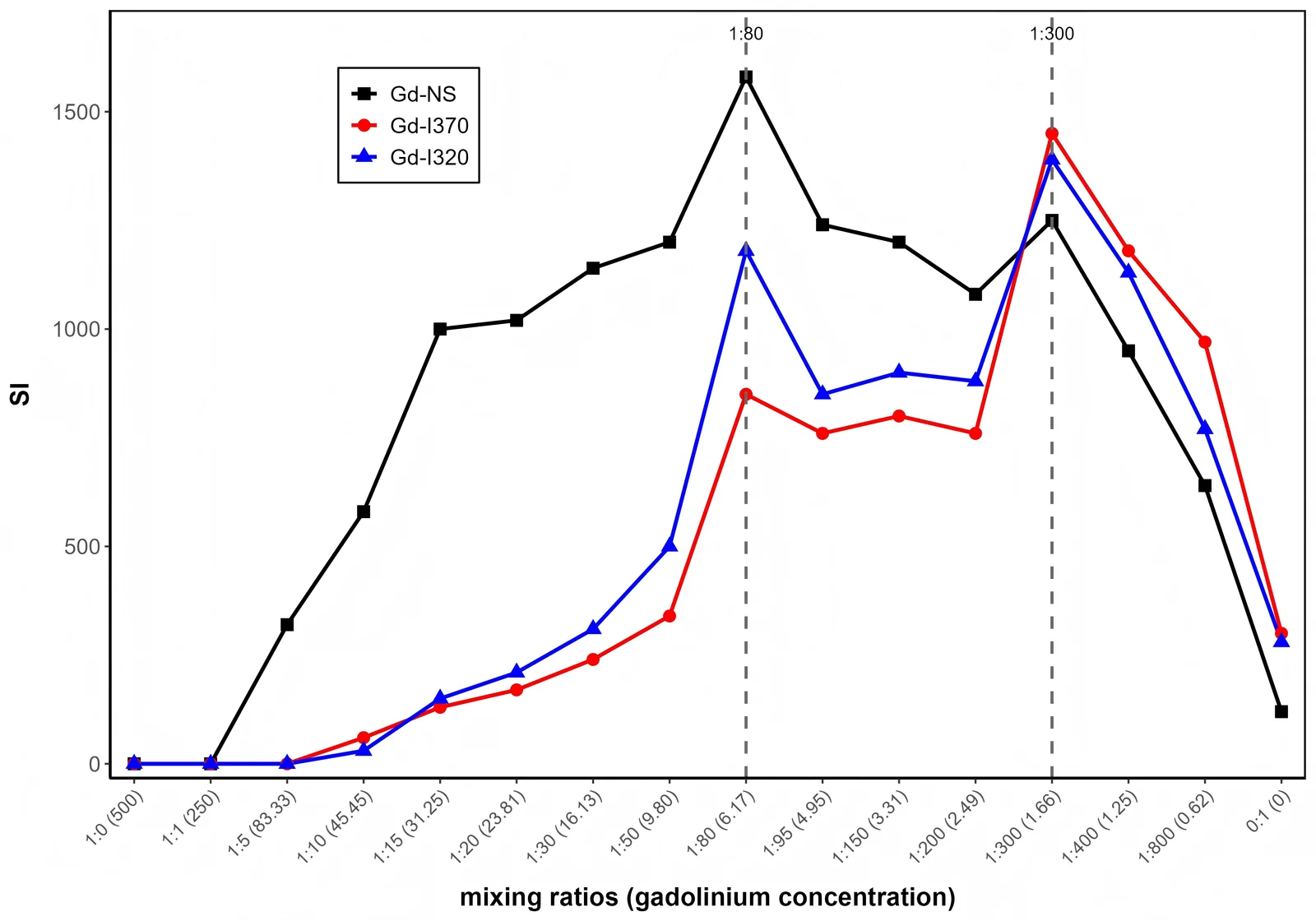
Signal intensity curves for three contrast agent mixtures at varying dilution ratios. Vertical dashed lines indicate the selected mixing ratios: 1:80 for Gd-NS (left) and 1:300 for both Gd-I370 and Gd-I320 (right). These ratios provided optimal signal intensity while maintaining practical viscosity for injection.

**Fig. (2):**
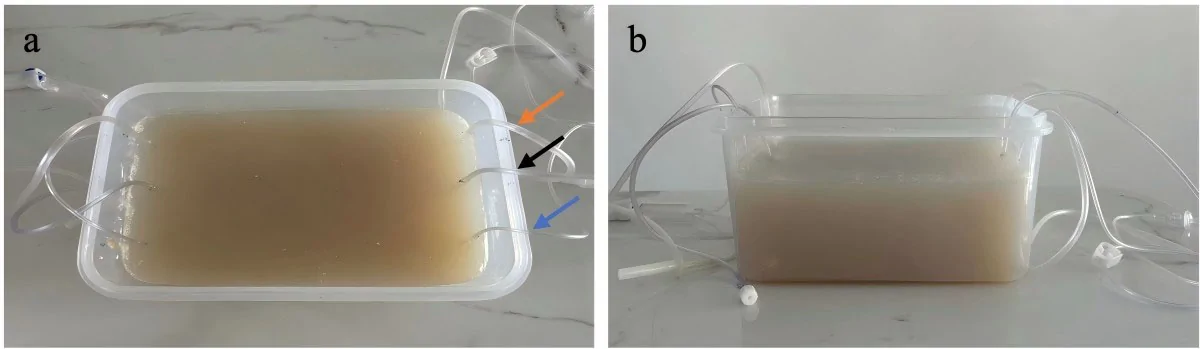
Phantom construction and experimental setup. (**A**) Cross-sectional view of a phantom showing three different inner diameter tubes embedded in agar gel matrix. Blue arrows indicate 4mm tubes, orange arrows 2mm tubes, and green arrows 1.4mm tubes. Scale bar = 2cm. (**B**) Complete phantom assembly with injection port (right) and drainage port (left) connected to infusion tubes. The temperature probe is visible for monitoring 37°C maintenance.

**Fig. (3):**
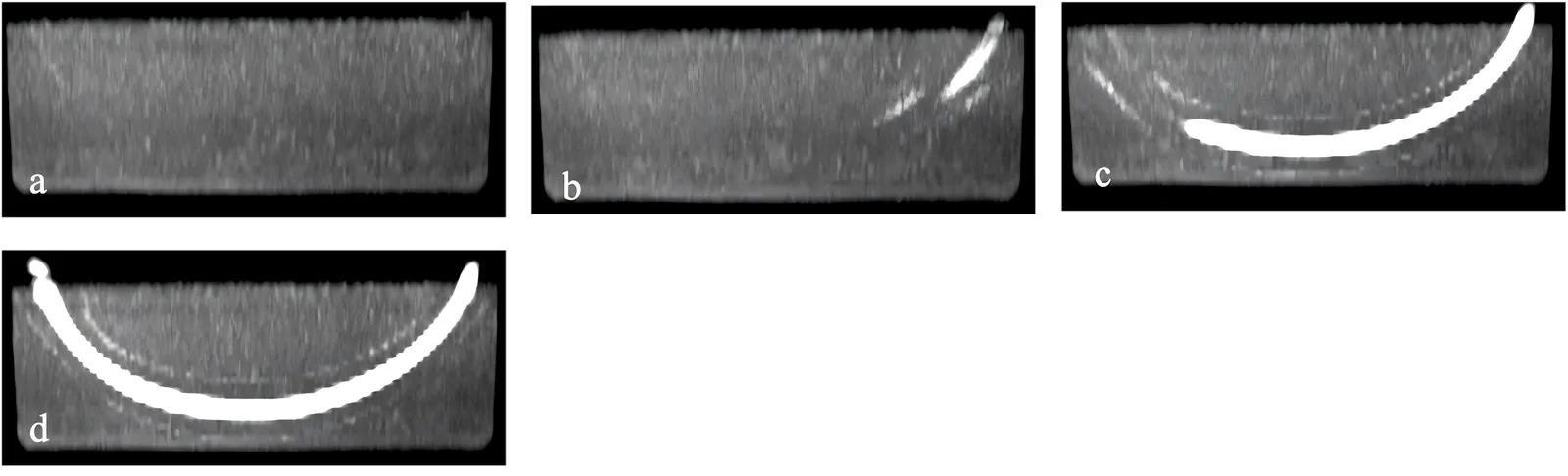
Dynamic contrast-enhanced imaging sequence showing the temporal progression of the contrast agent through a 4mm-diameter tube. (**a**) Baseline pre-contrast phase; (**b**) initial contrast entry at 5 seconds; (**c**) partial tube opacification at 15 seconds; (**d**) complete opacification at 40 seconds. Window width/level: 500/250.

**Fig. (4):**
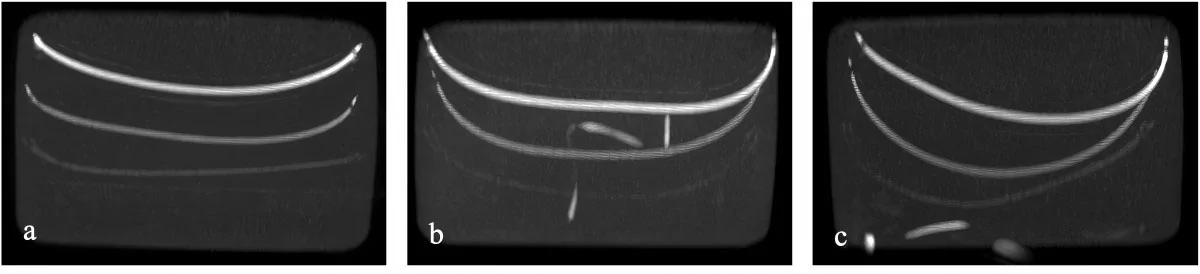
Maximum intensity projection (MIP) images comparing tube visualization across different diameters and contrast agents. Rows from top to bottom: 1.4mm, 2mm, and 4mm inner diameter tubes. Columns: (**a**) Gd-NS; (**b**) Gd-I370; (**c**) Gd-I320. The progressive improvement in signal intensity and homogeneity with increasing tube diameter, independent of contrast agent viscosity, can be noted.

**Fig. (5):**
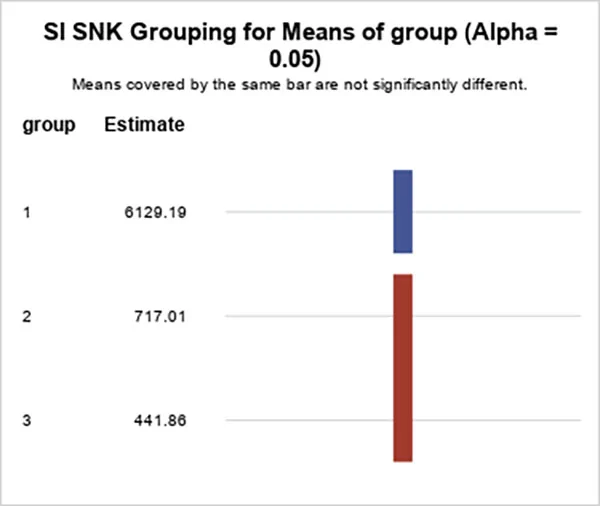
Statistical comparison of signal intensity by tube diameter. Groups 1, 2, and 3 represent 4mm, 2mm, and 1.4mm tubes, respectively. Horizontal bars indicate non-significant comparisons (none present - all comparisons *P*<0.001). Box plots show median, quartiles, and range.

**Fig. (6):**
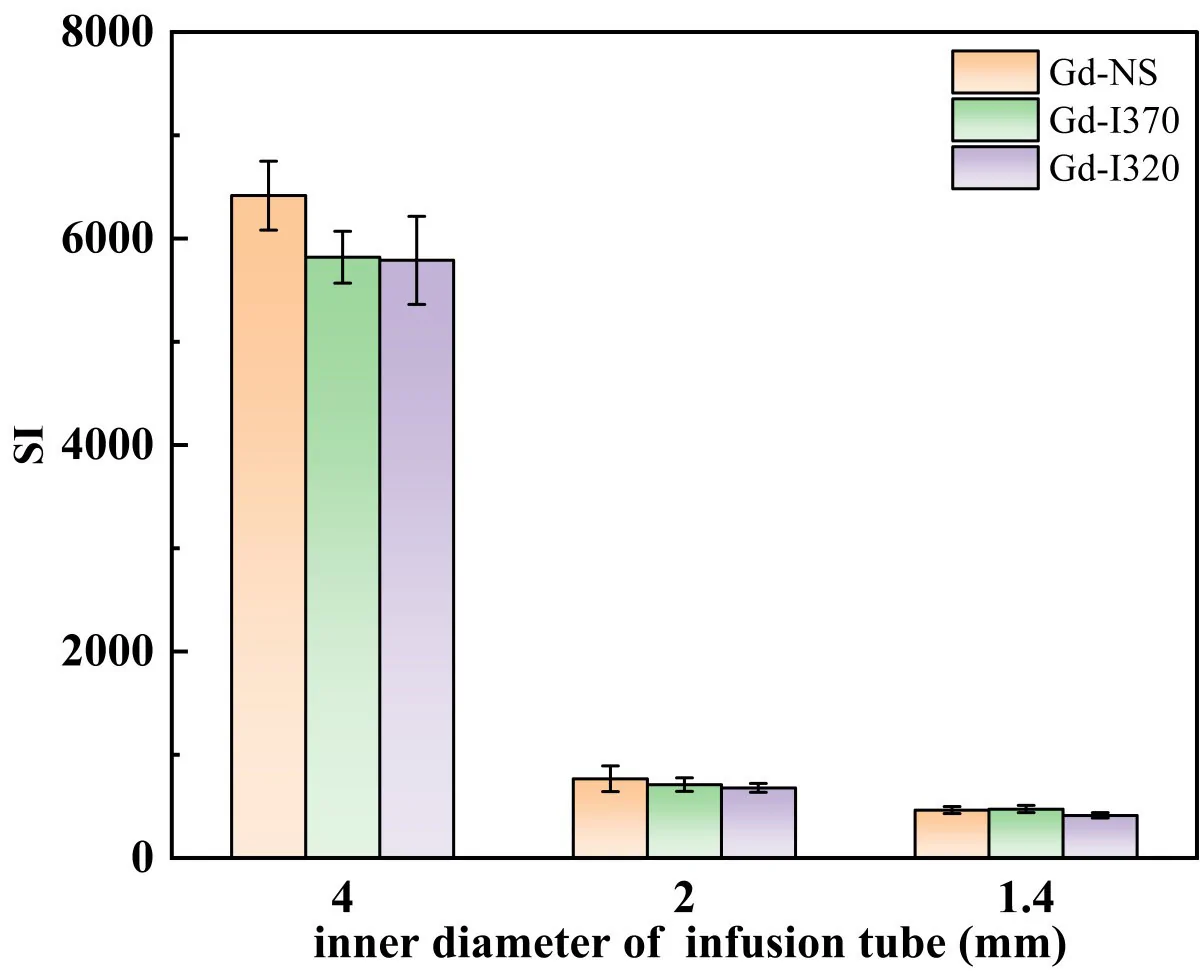
Signal intensity, signal-to-noise ratio (SNR), and contrast-to-noise ratio (CNR) as functions of tube inner diameter for all three contrast agents. Error bars represent the standard error of the mean. No significant differences have been found between contrast agents at any diameter (P>0.05).

**Table 1:** Viscosities of individual components and prepared contrast mixtures at different temperatures.

**Component/mixture**	**Viscosity (mPa·s)**	**-**
	20°C	37°C
**Individual components**		
Gadoterate meglumine injection	3.4	2.4
Iopromide injection (370 mgI/mL)	22.0	10.0
Iodixanol injection (320 mgI/mL)	25.4	11.4
Saline (0.9% NaCl)	1.0	0.7
**Prepared mixtures**		
1:80 Gd-NS	3.5 ± 0.1	2.6 ± 0.1
1:300 Gd-I370	12.8 ± 0.3	7.9 ± 0.2
1:300 Gd-I320	14.2 ± 0.3	8.7 ± 0.3

**Note:** Values for prepared mixtures represent mean ± SD from triplicate measurements.

**Table 2:** Signal intensity values for different contrast agents across phantom groups.

**Group**	**Signal intensity (mean ± SD)**			**H-value**	***P*-value**
	Phantom A	Phantom B	Phantom C		
Gd-NS (n=3)	2031.25 ± 2456	2049.71 ± 2478	2015.31 ± 2399	0.089	>0.05
Gd-I370 (n=3)	1989.14 ± 2421	2022.48 ± 2456	2001.66 ± 2411	0.089	>0.05
Gd-I320 (n=3)	2011.54 ± 2443	1998.82 ± 2402	2019.74 ± 2459	0.089	>0.05
Between groups				0.963	>0.05

**Table 3:** Signal intensity comparison by tube inner diameter.

**Inner diameter (mm)**	**Signal intensity (mean ± SD)**	**SNR**	**CNR**
4	5554.49 ± 1042	42.3 ± 8.1	38.7 ± 7.5
2	733.65 ± 78.76	12.4 ± 2.3	10.2 ± 1.9
1.4	444.55 ± 34.70	7.8 ± 1.2	5.9 ± 0.9

**Note:** F = 384.55, *P* < 0.001. All pairwise comparisons, *P* < 0.001. SNR: Signal-to-noise ratio; CNR: Contrast-to-noise ratio.

## Data Availability

The data and supportive information are available within the article.

## LIST OF ABBREVIATIONS

HSG: Hysterosalpingography
HyCoSy: Hysterosalpingo-contrast sonography
MR-HSG: Magnetic resonance hysterosalpingography
MIP: Maximum intensity projection
CNR: Contrast-to-noise ratio
SNR: Signal-to-noise ratio
ROIs: Regions of interest
